# Association of body mass index with severity and mortality of COVID-19 pneumonia: a two-center, retrospective cohort study from Wuhan, China

**DOI:** 10.18632/aging.202813

**Published:** 2021-03-24

**Authors:** Xiaodong Wu, Chenghong Li, Shi Chen, Xin Zhang, Feilong Wang, Ting Shi, Qiang Li, Lin Lin

**Affiliations:** 1 Department of Pulmonary and Critical Care Medicine, Shanghai East Hospital, Tongji University, Shanghai, China; 2 Department of Respiratory Medicine, The Sixth Hospital of Wuhan, Affiliated Hospital of Jianghan University, Wuhan, China; 3 Department of Pulmonary and Critical Care Medicine, People’s Liberation Army Joint Logistic Support Force 920th Hospital, Yunnan, China; 4 Centre for Global Health, Usher Institute, University of Edinburgh, Scotland, United Kingdom; 5 Department of Endocrine and Metabolic Diseases, Shanghai Institute of Endocrine and Metabolic Diseases, Ruijin Hospital, Shanghai Jiao Tong University School of Medicine, Shanghai, China; 6 Shanghai National Clinical Research Center for Metabolic Diseases, Key Laboratory for Endocrine and Metabolic Diseases of the National Health Commission of the PR China, Shanghai National Center for Translational Medicine, Ruijin Hospital, Shanghai Jiao Tong University School of Medicine, Shanghai, China

**Keywords:** COVID-19, SARS-CoV-2, body mass index, obesity, outcome

## Abstract

In this study, we aimed to investigate the relationship between body mass index (BMI) and multiple severe outcomes of the coronavirus disease 2019 (COVID-19) pneumonia. A total of 1091 patients hospitalized with COVID-19 pneumonia were included from Wuhan, China. Overall, 2.8% (n = 31) received invasive mechanical ventilation (IMV), 10.8% (n = 118) were admitted to the intensive care unit (ICU), 6.4% (n = 70) developed acute respiratory distress syndrome (ARDS), and 4.4% (n = 48) died. Multivariable-adjusted hazard ratios (HRs) (95% confidence intervals [CIs]) of IMV therapy, ICU admission and ARDS associated with obesity were 2.86 (1.16-7.05), 2.62 (1.52-4.49) and 3.15 (1.69-5.88), respectively; underweight was significantly associated with death (HR 3.85, 95%CI 1.26-11.76). Restricted cubic spline analyses suggested U-shaped associations of BMI with ICU admission and death, but linear relationships of BMI with IMV therapy and ARDS. In conclusion, obesity had an increased risk of IMV therapy, ICU admission and ARDS, while underweight was associated with higher mortality in COVID-19 pneumonia. U-shaped associations of BMI with ICU admission and death, and linear relationships of BMI with IMV therapy and ARDS, were found. These findings indicate that extra caution should be taken when treating COVID-19 patients with underweight and obesity.

## INTRODUCTION

The emerging coronavirus disease 2019 (COVID-19) first reported in Wuhan, China, is caused by a novel beta-coronavirus named severe acute respiratory syndrome coronavirus 2 (SARS-CoV-2) [1, 2]. It has rapidly spread across international borders causing a pandemic [3, 4]. The ongoing COVID-19 pandemic poses substantial challenges to global public health.

The clinical spectrum of patients with COVID-19 ranges from mild to severe illness [5, 6]. Risk factors associated with disease severity and outcome have been investigated in several studies [7, 8], indicating that elderly age was associated with higher risks of acute respiratory distress syndrome (ARDS) and death, and patients with co-morbidities, such as preexisting concurrent cardiovascular or cerebrovascular diseases, or ARDS were at an increased risk of death from COVID-19 pneumonia [11, 12].

So far, past studies and meta-analyses note that obesity could be a risk factor for severity of COVID-19. In a cohort study from US, BMI ≥ 30 kg/m^2^ was suggested as one of the most common comorbidities, accounting for 41.7% of total study population [3]. Among 4,103 patients with COVID-19 at an academic medical center in New York City, BMI >40 kg/m^2^ was the second strongest significant risk factor for hospitalization independently, after old age [9]. Another study from France found that the need for invasive mechanical ventilation (IMV) was related to a BMI ≥ 35 kg/m^2^ [10]. A meta-analysis including 30 studies indicated that obesity increased risks for hospitalization, ICU admission, IMV requirement and death among individuals with COVID-19 [11]. However, the effect of underweight on the outcomes of COVID-19 remained undetermined. Furthermore, most of those studies were conducted in American and European countries. Studies among Asian populations were sparse.

Therefore, we aimed to examine the association between BMI and multiple severe outcomes of COVID-19 pneumonia among Chinese patients, including IMV therapy, ICU admission, development of ARDS, and death.

## RESULTS

A total of 1091 patients hospitalized with COVID-19 pneumonia were included in this study. The median age was 59 years (IQR, 49-67 years), and 509 (46.7%) were men. Demographic and biochemical characteristics of the included patients are shown in Table 1, stratified by BMI categories. Overall, the median BMI was 22.51 kg/m^2^, with the underweight, normal weight, overweight and obesity accounting for 7.3%, 45.8%, 20.7% and 26.2% of the overall patients, respectively. The levels of neutrophil counts, lymphocyte counts, alanine transaminase (ALT), serum creatinine and hs-CRP increased with BMI (all p values for trend < 0.05). Of the 1091 patients, 430 (39.4%) patients had comorbidities, including diabetes (n = 137 [12.6%]), hypertension (n = 288 [26.4%]), CVD (n = 82 [7.5%]), CLD (n = 57 [5.2%]), and cancers (n = 40 [3.7%]). A total of 690 (63.2%) patients required oxygen support in the hospitals.

**Table 1:** Characteristics of patients hospitalized with COVID-19 pneumonia, stratified by BMI categories (n = 1091).

**Characteristics**	**Total**	**BMI categories**				
	**(n = 1091)**	**Underweight (n = 80)**	**Normal weight (n =500)**	**Overweight (n = 226)**	**Obesity (n = 285)**	***P* for trend**
Men, n (%)	509 (46.7)	15 (18.8)	168 (33.6)	132 (58.4)	194 (68.1)	< 0.0001
Age, years	59.00 (49.00-67.00)	64.00 (48.00-72.00)	59.50 (49.00-67.50)	59.00 (49.00-67.00)	58.00 (49.00-66.50)	0.24
BMI, kg/m^2^	22.51 (20.42-25.26)	17.81 (17.17-18.17)	20.76 (19.70-21.90)	24.22 (23.59-24.24)	27.04 (25.95-28.40)	< 0.0001
**Hematologic**						
Neutrophils, × 10^9^/L	3.52 (2.61-4.84)	3.33 (2.37-4.37)	3.40 (2.53-4.66)	3.52 (2.76-4.73)	3.72 (2.68-5.25)	0.0001
Lymphocytes, × 10^9^/L	1.23 (0.81-1.70)	1.28 (0.83-1.76)	1.28 (0.84-1.70)	1.24 (0.81-1.60)	1.11 (0.73-1.72)	0.02
Platelets, × 10^9^/L	210.00 (165.00-266.00)	208.50 (171.00-279.00)	211.00 (166.00-266.50)	215.50 (168.00-268.00)	202.00 (158.00-262.00)	0.23
**Biochemical**						
ALT, U/L	23.50 (15.54-37.40)	15.55 (10.80-23.13)	21.83 (14.35-35.60)	25.73 (17.39-40.96)	28.77 (18.20-44.10)	< 0.0001
Serum creatinine, umol/L	63.60 (53.00-76.00)	56.60 (50.85-63.80)	60.00 (52.20-72.20)	65.60 (53.10-77.90)	70.10 (59.90-81.60)	< 0.0001
hs-CRP, mg/L	7.58 (1.43-49.04)	4.21 (0.79-19.06)	4.97 (1.18-32.92)	16.99 (1.86-61.42)	20.14 (2.18-75.76)	< 0.0001
**Comorbidities**						
Diabetes, n (%)	137 (12.6)	7 (8.8)	62 (12.4)	33 (14.7)	35 (12.4)	0.53
Hypertension, n (%)	288 (26.4)	18 (22.5)	119 (23.8)	65 (28.8)	86 (30.2)	0.16
CVD, n (%)	82 (7.5)	2 (2.5)	40 (8.0)	14 (6.2)	26 (9.1)	0.22
CLD, n (%)	57 (5.2)	4 (5.0)	29 (5.8)	9 (4.0)	15 (5.3)	0.73
Cancer, n (%)	40 (3.7)	4 (5.0)	18 (3.6)	8 (3.5)	10 (3.5)	0.70
**Oxygen therapy**						
Nasal cannula, n (%)	607 (57.3)	55 (69.6)	283 (58.8)	122 (55.2)	147 (52.7)	0.009
NPPV, n (%)	176 (16.2)	11 (13.8)	55 (11.0)	43 (19.0)	67 (23.5)	< 0.0001
HFNC, n (%)	87 (8.0)	5 (6.3)	29 (5.8)	20 (8.9)	33 (11.6)	0.005
IMV, n (%)	31 (2.8)	0	10 (2.0)	4 (1.8)	17 (6.0)	0.0007

Data are n (%) or median (IQR). ALT, alanine transaminase. ARDS, acute respiratory distress syndrome. BMI, body mass index. CLD, chronic lung disease. COVID-19, coronavirus disease 2019. CVD, cardiovascular disease. HFNC, high flow nasal cannula. hs-CRP, high-sensitivity C-reactive protein. IMV, invasive mechanical ventilation. NPPV, non-invasive positive pressure ventilation.

**Figure 1:**
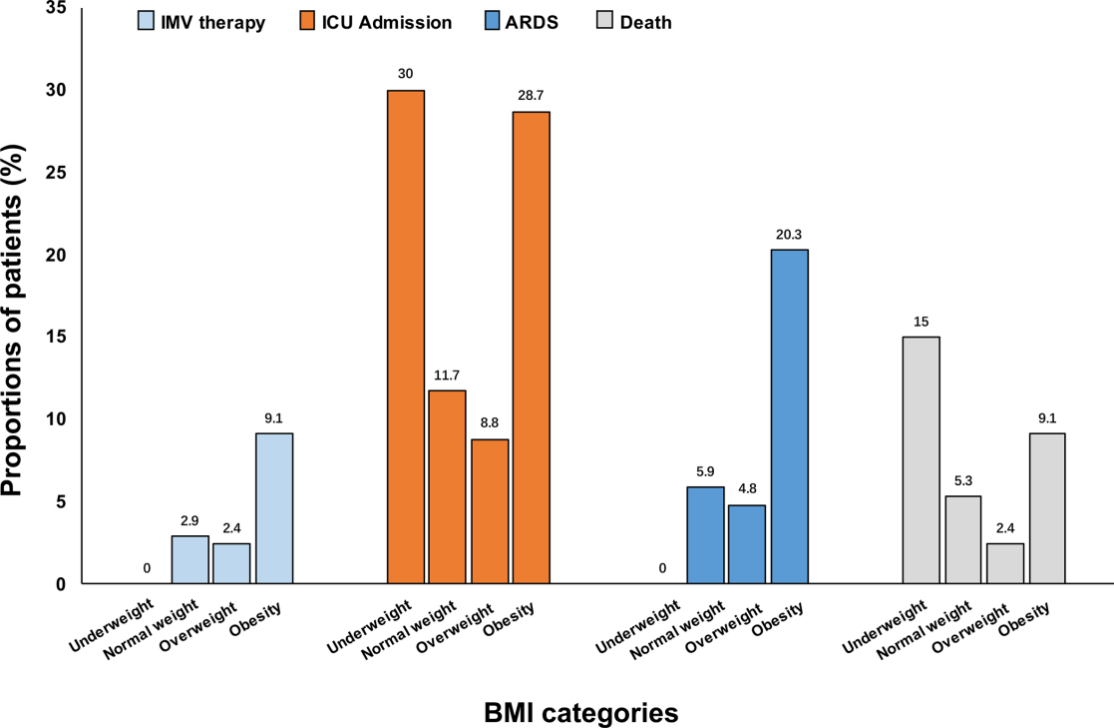
**Proportions of COVID-19 pneumonia patients with the outcomes according to BMI categories.** ARDS, acute respiratory distress syndrome. BMI, body mass index. COVID-19, coronavirus disease 2019. IMV, invasive mechanical ventilation. ICU, intensive care unit.

Of the entire cohort, 2.8% (n = 31) required IMV therapy, 10.8% (n = 118) were admitted to the ICU, 6.4% (n = 70) developed ARDS, and 4.4% (n = 48) died. Figure 1 showed the proportions of the clinical outcomes from patients with COVID-19 pneumonia by BMI category. Patients with obesity had higher rates of IMV therapy, ICU admission, ARDS and death, compared with the normal weight group. In reverse, the proportions of obesity in patients who required IMV therapy, who were admitted to ICU, who developed ARDS, and who died were higher, ranging from 33.3% to 55.7% (Supplementary Figure 2).

The characteristics of patients hospitalized with COVID-19 pneumonia were summarized in each category of clinical outcomes (IMV therapy, ICU admission, ARDS status and death) (Table 2). The levels of neutrophil counts, ALT, and hs-CRP were higher, while lymphocyte counts and platelet counts were lower in patients requiring IMV therapy (all p values *s* < 0.05), compared with those without IMV therapy. Patients requiring IMV therapy also had a higher proportion of comorbidities, including diabetes, CVD and cancers (all p values < 0.05). Similar estimates were observed for the other three clinical outcomes - ICU admission, ARDS, and death. Patients with these clinical outcomes also had an elevated rate of IMV therapy. Moreover, patients requiring IMV therapy, ICU admission and those with ARDS had increased BMI values than their respective counterparts (all p values < 0.05). There was no statistically significant difference in BMI between the death and survival groups.

**Table 2:** Characteristics of patients hospitalized with COVID-19 pneumonia, stratified by the outcome.

**Characteristics**	**IMV therapy**			**ICU admission**			**ARDS**			**Death**	
	**Not receiving IMV therapy (n = 1060)**	**Receiving IMV therapy (n = 31)**		**Without ICU admission (n = 973)**	**ICU admission (n = 118)**		**Without ARDS (n = 1021)**	**With ARDS (n = 70)**		**Alive (n = 1043)**	**Died (n = 48)**
Male, n (%)	486 (45.8)	22 (71.0) ^*^		435 (44.7)	74 (62.7) ^*^		461 (45.2)	48 (68.6) ^*^		481 (46.1)	28 (58.3) ^*^
Age, years	59.00 (49.00-67.00)	68.00 (62.00-86.00) ^*^		58.00 (48.00-66.00)	68.00 (58.00-80.00) ^*^		59.00 (49.00-67.00)	68.00 (57.00-82.00) ^*^		59.00 (49.00-67.00)	75.00 (65.50-85.50) ^*^
BMI, kg/m^2^	22.49 (20.42-25.01)	25.95 (22.49-28.69) ^*^		22.49 (20.38-24.84)	24.22 (20.76-27.43) ^*^		22.49 (20.31-24.91)	25.45 (22.49-28.39) ^*^		22.58 (20.42-25.26)	22.49 (19.48-26.00)
**Hematologic**											
Neutrophils, × 10^9^/L	3.50 (2.60-4.68)	5.87 (4.27-10.49) ^*^		3.42 (2.57-4.51)	5.43 (3.33-7.88) ^*^		3.46 (2.60-4.61)	5.51 (3.00-8.29) ^*^		3.46 (2.58-4.65)	5.51 (4.68-9.68) ^*^
Lymphocytes, × 10^9^/L	1.25 (0.82-1.71)	0.68 (0.45-0.83) ^*^		1.31 (0.89-1.74)	0.66 (0.46-0.84) ^*^		1.28 (0.86-1.72)	0.64 (0.45-0.79) ^*^		1.26 (0.83-1.71)	0.65 (0.45-0.87) ^*^
Platelets, × 10^9^/L	211.00 (167.00-268.00)	162.00 (129.00-186.00) ^*^		212.50 (168.00-268.00)	180.50 (137.00-234.00) ^*^		211.00 (168.00-268.00)	171.50 (139.00-223.00) ^*^		211.00 (167.00-266.50)	170.50 (126.00-223.50) ^*^
**Biochemical**											
ALT, U/L	23.30(15.41-37.13)	35.15(24.83-48.15) ^*^		23.15(15.35-36.30)	29.96(19.71-46.05) ^*^		23.35(15.43-36.90)	34.80(21.70-48.15) ^*^		23.40(15.40-37.00)	25.00(20.60-47.32) ^*^
Serum creatinine, umol/l	63.50(53.00-75.60)	72.12(56.22-105.90) ^*^		63.10(52.91-75.00)	69.23(58.73-90.70) ^*^		63.37(52.92-75.96)	66.55(59.41-85.04) ^*^		63.32(52.92-75.00)	84.10(61.51-112.18) ^*^
hs-CRP, mg/L	6.94(1.36-43.39)	86.43(58.45-138.20) ^*^		5.35(1.19-34.24)	80.37(43.32-116.06) ^*^		6.17(1.25-37.12)	84.32(62.71-112.90) ^*^		6.73(1.35-42.03)	92.31(43.89-149.37) ^*^
**Comorbidities**											
Diabetes, n (%)	128 (12.1)	8 (25.8) ^*^		111 (11.4)	26 (22.0) ^*^		123 (12.1)	14 (20.0)		124 (11.9)	13 (27.1) ^*^
Hypertension, n (%)	277 (26.1)	10 (32.3)		243 (25.0)	45 (38.1) ^*^		264 (25.9)	24 (34.3)		271 (26.0)	17 (35.4)
CVD, n (%)	75 (7.1)	6 (19.4) ^*^		62 (6.4)	20 (17.0) ^*^		70 (6.9)	12 (17.1) ^*^		75 (7.2)	7 (14.6)
CLD, n (%)	55 (5.2)	2 (6.5)		50 (5.1)	7 (5.9)		54 (5.3)	3 (4.3)		53 (5.1)	4 (8.3)
Cancer, n (%)	36 (3.4)	4 (12.9) ^*^		27 (2.8)	13 (11.0) ^*^		32 (3.1)	8 (11.4) ^*^		36 (3.5)	4 (8.3)
**Oxygen therapy**											
Nasal cannula, n (%)	597 (56.3)	7 (31.8) ^*^		572 (58.9)	35 (39.8) ^*^		601 (59.5)	6 (12.2) ^*^		589 (57.4)	18 (54.6) ^*^
NPPV, n (%)	167 (15.8)	9 (29.0) ^*^		106 (10.9)	70 (59.3) ^*^		138 (13.5)	38 (54.3) ^*^		152 (14.6)	24 (50.0) ^*^
HFNC, n (%)	77 (7.3)	10 (32.3) ^*^		48 (4.9)	39 (33.1) ^*^		62 (6.1)	25 (35.7) ^*^		76 (7.3)	11 (22.9) ^*^
IMV, n (%)	0	31		1 (0.1)	30 (25.4) ^*^		6 (0.6)	6 (12.2) ^*^		12 (1.2))	19 (39.6) ^*^

Data are n (%) or median (IQR). ALT, alanine transaminase. ARDS, acute respiratory distress syndrome. BMI, body mass index. CLD, chronic lung disease. COVID-19, coronavirus disease 2019. CVD, cardiovascular disease. HFNC, high flow nasal cannula. hs-CRP, high-sensitivity C-reactive protein. IMV, invasive mechanical ventilation. NPPV, non-invasive positive pressure ventilation. ^*^ *P* < 0.05

We further investigated the potential risk factors associated with IMV therapy and other clinical outcomes of COVID-19 pneumonia (Supplementary Table 1). Multivariate Cox regression analyses revealed that age ≥ 60 years, men, neutrophil counts, lymphocyte counts, platelet counts, hs-CRP and cancer were independently associated with IMV therapy, ICU admission and ARDS. Several above-mentioned risk factors (men, platelet counts and cancer) were not associated with death. Each 1 kg/m^2^ increment of BMI was found to be related to IMV therapy and ARDS, but not to ICU admission and death.

**Figure 2:**
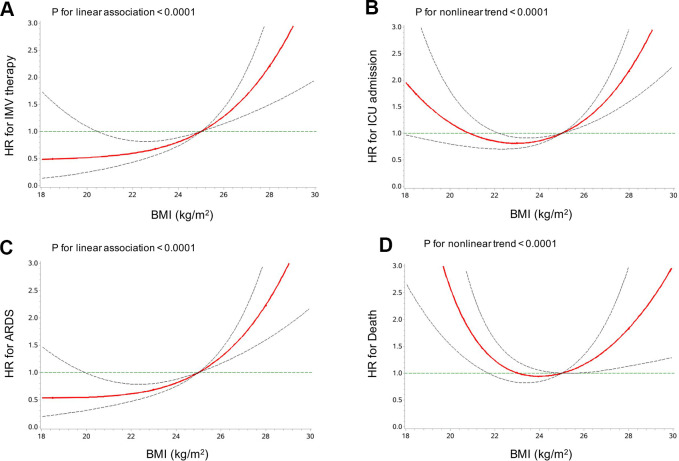
Multivariable-adjusted HRs (95% CIs) for the associations of IMV therapy (**A**), ICU admission (**B**), ARDS (**C**) and death (**D**) with BMI. HRs (95% CIs) were adjusted for age, sex, neutrophil counts, lymphocyte counts, platelet counts, hs-CRP, and cancer (yes/no). ARDS, acute respiratory distress syndrome. BMI, body mass index. ICU, intensive care unit. IMV, invasive mechanical ventilation. Red line, HR; dotted grey lines, 95% CI.

The risk of requiring IMV therapy and other clinical outcomes of COVID-19 pneumonia according to BMI categories was further analyzed. As demonstrated in Table 3, compared with patients in the normal weight group, those obese had an increased risk of requiring IMV therapy (HR 2.86, 95% CI 1.16-7.05), ICU admission (HR 2.62, 95% CI 1.52-4.49) and developing ARDS (HR 3.15; 95% CI 1.69-5.88) in the multivariable-adjusted model. However, the association of obesity and with death was not significant in the adjusted model. However, underweight was associated with a 2.85-fold higher risk of death after adjusting for potential confounders (HR 3.85, 95% CI 1.26-11.76). The test for interaction of age, hypertension and cancer, with obesity on the risk of IMV therapy, ICU admission and ARDS, was statistically significant (not for other stratified variables or death, all p-values for interaction < 0.05, data not shown). When we further adjusted diabetes (yes/no), hypertension (yes/no), CVD (yes/no), and center, the results did not change appreciably (Supplementary Table 2). Multivariable-adjusted restricted cubic spline analyses suggested “U-shaped” associations between BMI and both ICU admission and death of COVID-19 pneumonia, which indicated significant nonlinear relationships (both *P* < 0.0001) (Figure 2). There was evidence of a significant linear relationship of BMI with IMV therapy and ARDS (both *P* < 0.0001).

**Table 3:** The association between BMI category and outcome in patients with COVID-19 pneumonia.

	**BMI subgroups**			
	**Underweight**	**Normal weight**	**Overweight**	**Obesity**
**IMV therapy**				
Model 1	NA	1.00	0.88 (0.27-2.83)	**3.11 (1.40-6.88)**
Model 2	NA	1.00	0.85 (0.24-2.98)	**2.86 (1.16-7.05)**
**ICU admission**				
Model 1	1.94 (0.95-3.97)	1.00	0.93 (0.51-1.70)	**2.78 (1.78-4.34)**
Model 2	2.18 (0.94-5.08)	1.00	0.84 (0.42-1.68)	**2.62 (1.52-4.49)**
**ARDS**				
Model 1	0.28 (0.04-2.07)	1.00	0.80 (0.35-1.82)	**3.44 (2.00-5.94)**
Model 2	0.23 (0.03-1.85)	1.00	0.71 (0.29-1.73)	**3.15 (1.69-5.88)**
**Death**				
Model 1	**3.71 (1.27-10.79)**	1.00	0.57 (0.15-2.10)	**2.50 (1.08-5.79)**
Model 2	**3.85 (1.26-11.76)**	1.00	0.53 (0.14-2.00)	1.75 (0.73-4.21)

Model 1: unadjusted.

Model 2: adjusted for age, sex, neutrophil counts, lymphocyte counts, platelet counts, hs-CRP, and cancer (yes/no).

ARDS, acute respiratory distress syndrome. BMI, body mass index. HR, hazard ratio. ICU, intensive care unit. IMV, invasive mechanical ventilation. HR, hazard ratio. NA, not available.

## DISCUSSION

In this cohort study, we investigated the associations of BMI with multiple severe outcomes in patients with COVID-19 pneumonia, including IMV therapy, ICU admission, development of ARDS, and death. We found that patients with obesity had an increased risk of IMV therapy, ICU admission and developing ARDS. In addition, underweight was independently associated with death. Our data further suggested U-shaped associations of BMI with ICU admission and death. However, a linear relationship was detected for BMI with IMV therapy and ARDS. These findings suggest that patients with obesity or underweight were at higher risks of severe outcomes from COVID-19 pneumonia and thus extra caution should be taken when treating COVID-19 patients with underweight and obesity.

The COVID-19 pandemic has led to the global research efforts to identify individuals at the greatest risk of developing critical illness, including death. Previous studies have shown that older individuals are particularly vulnerable, as well as those with metabolic comorbidities [8, 12]. Evidence has recently emerged regarding the association between a higher prevalence of obesity among COVID-19 patients and an increased risk of poor prognosis from SARS-CoV-2 infection. A descriptive study with 24 critically ill patients diagnosed with COVID-19 in the Seattle region was among the first to report BMI data [13], showing that 85.0% of the patients with obesity required mechanical ventilation and 62.0% of the patients with obesity died. These proportions were greater than those in the patients without obesity. In a latest cohort study from the US with more than 5700 patients [9], obesity (BMI ≥ 30 kg/m^2^) was suggested as one of the most common comorbidities, accounting for 41.7% of the total study population. In our study, we found that among patients who required IMV therapy, were admitted to ICU, developed ARDS, and those who died, the prevalence rates of obesity were 61.9%, 52.6%, 64.4% and 46.4%, respectively (data not shown). We also reported higher proportions of receiving IMV therapy, requirement of ICU admission, development of ARDS and death in obese patients than those with normal weight (unadjusted), which was consistent with the published data.

In a small study consisting of 124 patients with COVID-19 from France [10], the need for IMV therapy was found to be associated with a BMI ≥ 35 kg/m^2^, independently from other comorbidities. Data from Shenzhen, China with 383 COVID-19 patients revealed that overweight was associated with an 86% higher, and obesity with a 142% higher, risk of developing severe pneumonia compared with patients with normal weight [14]. A recent study with 214 patients from Wenzhou [15], China reported a 6-fold increased risk of severe COVID-19 illness, but with a large 95% confidence intervals. Our study showed an increased risk of IMV therapy, ICU admission and developing ARDS (adjusted) among obese patients, using the World Health Organization recommendations for Asian populations as BMI ≥ 25 kg/m^2^ to define obesity.

Multiple studies have observed the associations between obesity and death with COVID-19, which was not found significant in our study after adjusting for other variables. One possible reason could be that the prevalence of obesity, especially severe obesity in Chinese population is extremely lower than that in western countries. As evidenced in recent data, the prevalence of obesity is 6.2% in China, versus 40% in the USA, 20% in Italy, and 24% in Spain [16]. Besides, many studies demonstrated that the excess risk from obesity on death was limited mostly to the younger patients with class III obesity (BMI ≥ 40 kg/m^2^). Tartof et al reported that a higher BMI (≥ 40 kg/m^2^) was associated with an increased risk of death from COVID-19 [17], particularly in male patients and younger individuals (≤60 years), in both outpatients and inpatients. S. Hendren et al found that the association of BMI with death or mechanical ventilation was strongest in adults ≤50 years [18]; severe obese individuals (BMI ≥40 kg/m^2^) had an increased risk of in-hospital death, only in those ≤50 years (hazard ratio, 1.36 [1.01–1.84]). However, the number of patients with BMI ≥ 40 kg/m^2^ was sparse in our study, which might explain why the adjusted effect of obesity on death was not identified in the current study.

From an indirect perspective, obesity and excess fat mass are commonly related to other comorbidities, such as hypertension, diabetes, CVD and renal disease, which are considered to result in increased vulnerability to pneumonia-associated organ failures [19]. For example, in individuals with diabetes, obesity and excess ectopic fat lead to impairment of beta-cell function and increased insulin resistance. Therefore, an appropriate metabolic response upon immunologic challenge was limited, resulting in some diabetes patients requiring substantial amounts of insulin during severe infections [20]. Patients with obesity often have respiratory dysfunction, which is characterized by alterations in respiratory mechanisms, increased airway resistance, impaired gas exchange, diminishing forced volume and forced vital capacity, possibly leading to ARDS. With respect to the immune response, there is a clear association between obesity and basal inflammatory status characterized by higher circulating Interleukin 6 and CRP levels. Actually, adipose tissue in obesity is “pro-inflammatory”, with increased expression of cytokines and particularly adipokines. Obesity was reported to impair adaptive immune responses to influenza virus [21], and conceivably could do so in COVID-19. Our study showed higher levels of hs-CRP in obese patients than those with normal weight, indicating strong systemic inflammation.

In this study, underweight was significantly associated with an increased risk of death, and a U-shaped association between BMI and death was identified. That is, both underweight and obesity showed increased trend and risk of death due to COVID-19, compared with the normal weight. In a retrospective cohort study with 1687 hospitalized patients, being underweight had a statistically significant association with an increased risk for death (HR=2.37, 95%CI 1.50–3.75) [22], which was consistent with our findings. In another study with 10,861 patients with COVID-19 infection, being underweight was suggested to have a non-significant association with IMV, but have a higher risk of death (OR = 1.44, 95% CI 1.08-1.92) [23]. It can be speculated that being underweight is often associated with malnutrition, impaired immune function, underlying frailty along with coexisting chronic conditions. Furthermore, being underweight is at an increased risk for pneumonia, and worse outcomes among older hospitalized patients [24].

To the best of our knowledge, this was the one of the limited studies among Asian population to investigate the association of BMI and multiple severe outcomes of COVID-19 pneumonia. Our present study was subjected to some limitations. First, the number of cases of IMV therapy outcome in the underweight group was too small to perform the analysis, which could reduce the robust of the analysis result. Second, although most height and weight values were retrieved from medical chart records, some were self-reported, which may lead to measurement bias. Third, even though potential influencing factors had been adjusted, our results might still be confounded by other factors that are unavailable in current study, such as lifestyle information and therapeutic agents use. Fourth, the definition of obesity (BMI ≥25 kg/m^2^) in this study was appropriate for Asians, so comparability of the findings with other studies which have used the BMI cut-off of 30 kg/m^2^ is compromised.

In conclusion, our study suggested that patients with underweight or obesity experienced more severe outcomes than those of normal weight when being hospitalized with COVID-19 pneumonia. Our findings highlight the significant role of BMI in clinical progression of COVID-19 pneumonia, and this should be kept in mind when treating COVID-19 patients with underweight and obesity.

## MATERIALS AND METHODS

### Study population

This two-center, retrospective cohort study was conducted at the Sixth Hospital of Wuhan and Taikang Tongji Hospital of Wuhan, which were the two government-designated hospitals for the patients with COVID-19. The cohort consisted of 1171 adult patients aged 21 to 93 years old with confirmed COVID-19 pneumonia who were admitted between January 1 to March 1, 2020 and who died or were discharged before March 30, 2020. COVID-19 pneumonia was diagnosed according to World Health Organization (WHO) interim guidance [25]. In our study, we excluded patients with missing data of BMI (n = 58). We further excluded 22 patients who didn’t have data on the outcomes (IMV therapy, ICU admission, development of ARDS, or death), resulting in 1091 patients for this analysis (Supplementary Figure 1). The ethics committee of the Sixth Hospital of Wuhan and Shanghai East Hospital approved this study and empowered a waiver of informed consent from study participants.

### Data collection

Patients’ demographic characteristics data, such as age and sex, were extracted from the electronic medical records. The data on the underlying comorbidities were extracted from patients’ reports of past medical history, including diagnosed type 2 diabetes mellitus (T2DM), hypertension, cardiovascular disease (CVD), chronic lung disease (CLD), and cancer. Blood tests on admission were extracted from the electronic medical records including hematologic and biochemical indexes. A standardized data collection form was used by a team of experienced respiratory clinicians. Data were double checked by a third reviewer if there was a disagreement. Anthropometric measurements, including body weight and height were performed by trained nurses according to standard protocols. BMI (kg/m^2^) was calculated by dividing weight (kg) by height (m) squared. The majority of the BMI data (n = 1048) used in this study were calculated based on the measurements on the first day of hospital admission, and the rest of BMI data (n = 43) were calculated according to self-reported height and weight values obtained from the patients and their family members.

For the diagnosis of SARS-CoV-2 infection, plasma and nasopharyngeal swab samples were obtained from all patients upon hospital admission and tested for targeted NP gene segment by real-time reverse transcription polymerase chain reaction (RT-PCR) assays as described elsewhere [1]. The requirement of oxygen therapy during hospitalization (nasal cannula, noninvasive positive pressure ventilation [NPPV], high flow nasal cannula oxygen therapy (HFNC), or invasive mechanical ventilation [IMV]) was recorded during hospitalisation.

### Assessment of obesity

BMI was categorized by the definitions as follows: 1) underweight (BMI < 18.5kg/m^2^); 2) normal weight (BMI 18.5–23 kg/m^2^); 3) overweight (BMI 23– 25 kg/m^2^); 4) obesity (BMI ≥ 25 kg/m^2^) according to the World Health Organization recommendations for Asian populations [26]. The validity of this definition has been confirmed previously [27, 28].

### Follow-up and outcome assessment

The information on clinical outcomes was obtained from the electronic medical records and verified by two authors (C.H.L and X.D.W). The investigated outcomes included requirement of IMV therapy, ICU admission, development of ARDS, and death. The definition of ARDS was according to the interim guidance of WHO for SARS-CoV-2 [25]. Briefly, the definition criteria for ARDS were as follows: acute onset within 1 week of new or worsening respiratory symptoms; the radiographic images showing bilateral opacities not fully explained by lobar or lung collapse, effusions, or nodules; the oxygenation was impaired as measured by a PaO_2_/FiO_2_ (fraction of inspired oxygen) not exceeding 300 mmHg with a minimal positive end-expiratory pressure level of 5 cm H_2_O.

### Statistical analysis

Demographic and biochemical characteristics from the included patients were summarized as medians (interquartile ranges [IQRs]) for the continuous variables and numbers (proportions) for the categorical variables, stratified by BMI category, and by clinical outcome (IMV therapy, ICU admission, ARDS and death) respectively. The differences across the BMI categories or the clinical outcomes were determined by one-way ANOVA (for continuous variables) or the χ^2^ test (the categorical variables). We tested the p values for trend through the BMI categories treating BMI as an ordinal variable.

In order to analyze the associations of BMI with clinical outcomes (IMV therapy, ICU admission, ARDS and death), univariable and multivariable Cox proportional hazards models were performed. Proportional hazards assumption was verified by regressing Schoenfeld residuals over time. Hazard ratios (HRs) and the corresponding 95% confidence intervals (CIs) for potential risk factors and each BMI subgroup in relation to these events were further evaluated, using the normal BMI (BMI 18.5–23kg/m^2^) as a reference. In the time-to-event analysis, data were censored at the time of death or the discharge-whichever occurred first. Covariates in the multivariate-adjusted models include age, sex, neutrophil counts, lymphocyte counts, platelet counts, high-sensitivity C-reactive protein (hs-CRP), and cancer (yes/no). The p values for interaction were calculated by a likelihood ratio test comparing models with and without the interaction terms.

We examined the potential non-linear associations between the level of BMI and the incidence of IMV therapy and other clinical outcome using restricted cubic splines. Analyses were multivariate-adjusted, using three knots, which were located at the 5th, 50th and 95th percentiles for baseline BMI levels, and the highest and lowest 0.5% were trimmed. Tests for non-linearity, which compared a model containing only the linear term with a model containing the linear and restricted cubic spline terms, were performed using likelihood ratio tests. If a test for non-linearity was not significant, a test for linearity was performed, comparing a model containing the linear term with a model containing only the covariates of interest.

PASS 15 software was used to test the statistical power. The effect size we anticipated was at least 1.49 based on the previous studies [11]. It is suggested that we had a 99% chance of detecting a 49% increased risk of IMV therapy, ICU admission, development of ARDS, and death in patients with obesity at an alpha level of 0.05 in the present study. Significance tests were two-tailed, with a p value < 0.05 considered as statistically significant. Statistical analysis was performed using SAS version 9.4 (SAS Institute, Cary, NC, USA).

### Data availability statement

All data used during the study are available from the corresponding author by request.

## Supplementary Material

Supplementary Figures

Supplementary Tables
