# Supplementary material for: Association of body mass index with severity and mortality of COVID-19 pneumonia: a two-center, retrospective cohort study from Wuhan, China
Source: Aging (Albany NY). 2021 Mar 24;13(6):7767–80. doi: 10.18632/aging.202813 (PMC8034951; doi:10.18632/aging.202813)
Supplement: Supplementary Figures [file aging-13-202813-s001.pdf]

SUPPLEMENTARY FIGURES

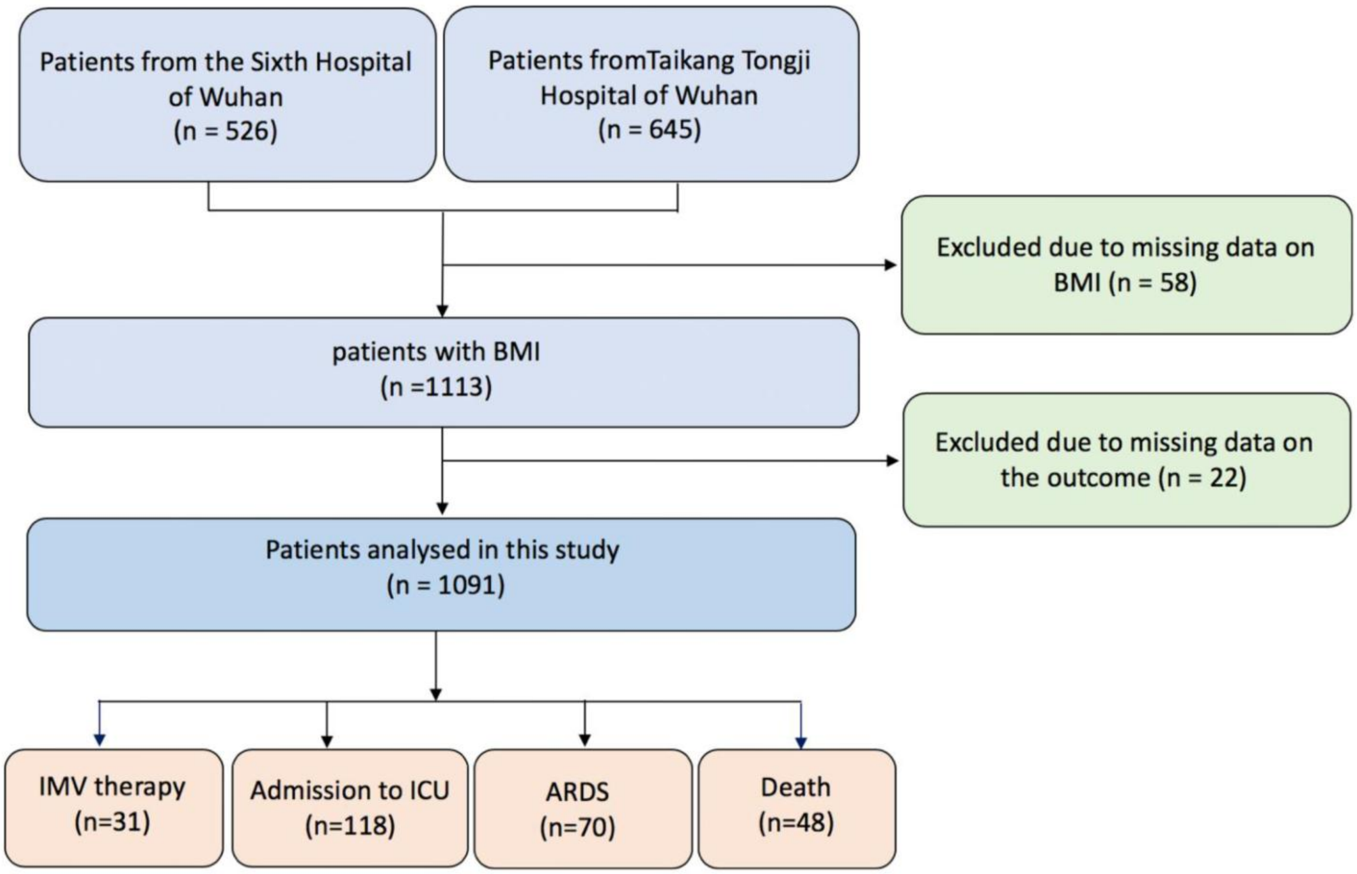

Supplementary Figure 1. Flowchart of study population. BMI, body mass index.

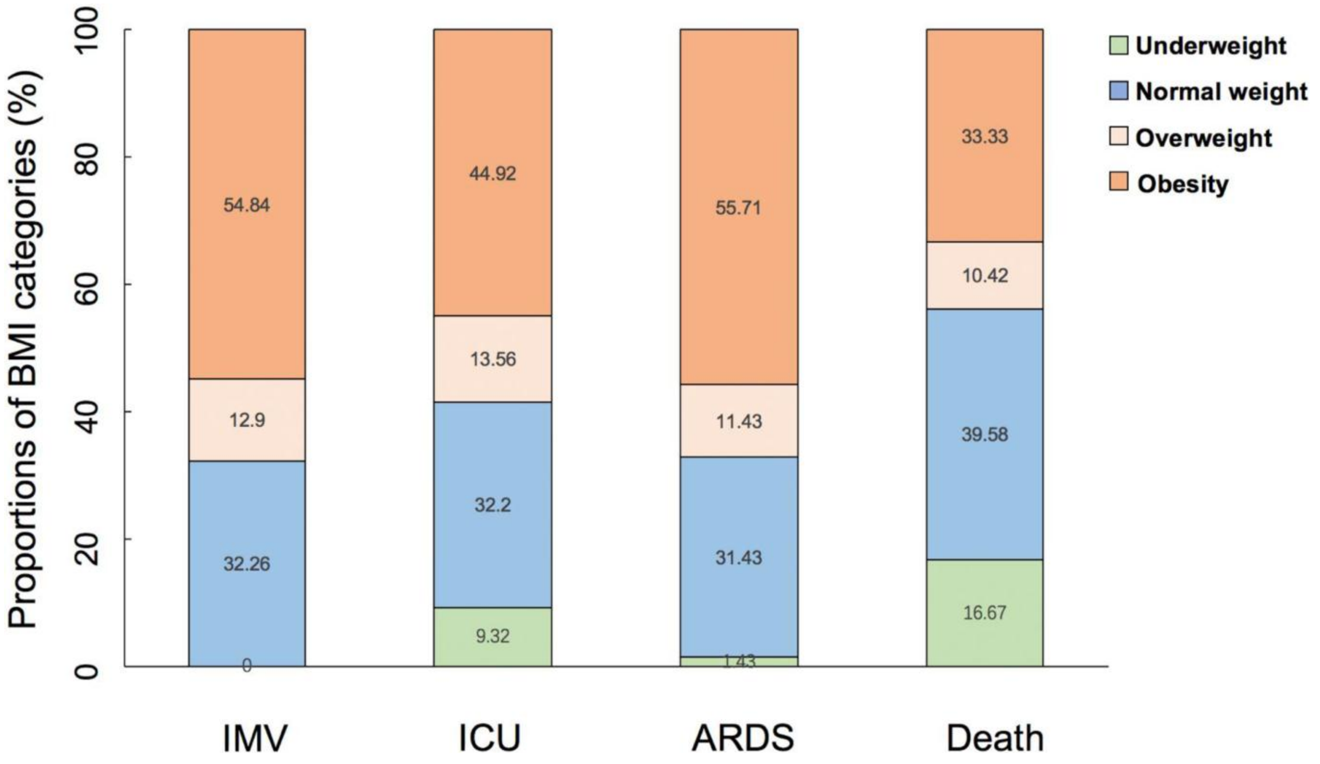

Supplementary Figure 2. Proportions of BMI categories according to the outcomes of COVID-19 pneumonia. ARDS, acute respiratory distress syndrome. BMI, body mass index. COVID-19, coronavirus disease 2019. IMV, invasive mechanical ventilation. ICU, intensive care unit.
