# Supplementary material for: Association of body mass index with severity and mortality of COVID-19 pneumonia: a two-center, retrospective cohort study from Wuhan, China
Source: Aging (Albany NY). 2021 Mar 24;13(6):7767–80. doi: 10.18632/aging.202813 (PMC8034951; doi:10.18632/aging.202813)
Supplement: Supplementary Tables [file aging-13-202813-s002.pdf]

SUPPLEMENTARY TABLES

Supplementary Table 1. Univariable and multivariable Cox regression of risk factors associated with outcome in patients with COVID-19 pneumonia.

	IMV therapy		ICU admission		ARDS		Death	
	Model 1	Model 2	Model 1	Model 2	Model 1	Model 2	Model 1	Model 2
Sex (male vs female)	**2.87 (1.31-6.29)**	**2.93 (1.33-6.45)**	**2.08 (1.40-3.08)**	**2.15 (1.44-3.20)**	**2.65 (1.58-4.46)**	**2.71 (1.61-4.57)**	1.52 (0.76-3.06)	1.51 (0.75-3.04)
Age (≥60 vs < 60), years	**3.60 (1.54-8.43)**	**3.67 (1.57-8.62)**	**3.05 (1.99-4.66)**	**3.12 (2.03-4.78)**	**2.69 (1.58-4.58)**	**2.75 (1.61-4.70)**	**3.34 (1.37-8.15)**	**3.33 (1.36-8.12)**
BMI, kg/m^2	**1.17 (1.07-1.28)**	**1.19 (1.07-1.31)**	**1.11 (1.05-1.17)**	1.05 (0.97-1.11)	**1.21 (1.13-1.29)**	**1.21 (1.13-1.30)**	1.00 (0.91-1.10)	1.01 (0.91-1.12)
**Hematologic**								
Neutrophils, × 10^9/L	**1.16 (1.07-1.25)**	**1.14 (1.06-1.22)**	**1.26 (1.19-1.34)**	**1.24 (1.17-1.33)**	**1.18 (1.11-1.26)**	**1.16 (1.09-1.24)**	**1.12 (1.08-1.16)**	**1.13 (1.09-1.17)**
Lymphocytes, × 10^9/L	**0.05 (0.02-0.16)**	**0.08 (0.03-0.23)**	**0.08 (0.04-0.13)**	**0.09 (0.05-0.16)**	**0.07 (0.03-0.13)**	**0.08 (0.04-0.17)**	**0.18 (0.07-0.45)**	**0.22 (0.08-0.56)**
Platelets, × 10^9/L	**0.989 (0.983-0.995)**	**0.991 (0.985-0.996)**	**0.996 (0.993-0.998)**	**0.997 (0.994-0.999)**	**0.994 (0.991-0.998)**	**0.995 (0.992-0.999)**	1.00 (0.99-1.01)	0.99 (0.98-1.00)
**Biochemical**								
ALT, U/L	**1.00 (1.00-1.02)**	1.00 (0.99-1.02)	**1.01 (1.00-1.02)**	0.99 (0.97-1.01)	**1.008 (1.001-1.015)**	1.00 (0.99-1.01)	**1.01 (1.00-1.02)**	1.00 (0.99-1.02)
Serum creatinine, umol/l	**1.003 (1.000-1.007)**	1.00 (0.99-1.01)	**1.01 (1.00-1.02)**	1.00 (0.99-1.03)	1.00 (0.99-1.01)	1.00 (0.99-1.01)	**1.01 (1.00-1.02)**	1.00 (0.99-1.02)
hs-CRP, each 10 increment, mg/L	**1.19 (1.13-1.26)**	**1.17 (1.11-1.24)**	**1.21 (1.16-1.25)**	**1.18 (1.14-1.23)**	**1.12 (1.06-1.18)**	**1.17 (1.12-1.22)**	**1.20 (1.13-1.27)**	**1.18 (1.12-1.25)**
**Comorbidities**								
Diabetes (yes vs no)	**2.51 (1.10-5.74)**	1.92 (0.83-4.46)	**2.19 (1.36-3.53)**	**1.73 (1.06-2.84)**	1.82 (0.98-3.37)	1.43 (0.76-2.69)	2.14 (0.96-4.77)	1.83 (0.81-4.11)
Hypertension (yes vs no)	1.34 (0.62-2.88)	0.96 (0.44-2.10)	**1.85 (1.24-2.76)**	1.41 (0.93-2.13)	1.50 (0.90-2.50)	1.16 (0.42-1.72)	1.67 (0.80-3.49)	1.25 (0.59-2.65)
CVD (yes vs no)	**3.14 (1.25-7.89)**	2.12 (0.82-5.50)	**3.00 (1.74-5.18)**	**2.11 (1.20-3.77)**	**2.81 (1.44-5.48)**	**2.03 (1.01-4.07)**	1.72 (0.60-4.92)	1.29 (0.45-3.71)
CLD (yes vs no)	1.26 (0.29-5.40)	0.83 (0.19-3.65)	1.16 (0.52-2.63)	0.82 (0.36-1.89)	0.80 (0.24-2.63)	0.55 (0.17-1.85)	1.94 (0.59-6.39)	1.54 (0.46-5.12)
Cancer (yes vs no)	**4.20 (1.40-12.63)**	**3.55 (1.14-11.05)**	**4.34 (2.17-8.66)**	**3.94 (1.90-8.15)**	**3.99 (1.76-9.02)**	**3.64 (1.55-8.54)**	2.50 (0.84-7.46)	2.11 (0.71-6.29)

Model 1: unadjusted.
Model 2: adjusted for age and sex.
ALT, alanine transaminase. ARDS, acute respiratory distress syndrome. CLD, chronic lung disease. BMI, body mass index. CLD, chronic lung disease. CVD, cardiovascular disease. HR, hazard ratio. hs-CRP, high-sensitivity C-reactive protein. ICU, intensive care unit. IMV, invasive mechanical ventilation. OR, odds ratio.

Supplementary Table 2. The association between BMI category and outcome in patients with COVID-19 pneumonia.

	BMI subgroups			
	**Underweight**	**Normal weight**	**Overweight**	**Obesity**
**IMV therapy**				
Model 1	NA	1.00	0.88 (0.27-2.83)	**3.11 (1.40-6.88)**
Model 2	NA	1.00	0.85 (0.24-2.98)	**2.85 (1.15-7.05)**
**ICU admission**				
Model 1	1.94 (0.95-3.97)	1.00	0.93 (0.51-1.70)	**2.78 (1.78-4.34)**
Model 2	2.17 (0.94-5.05)	1.00	0.84 (0.42-1.68)	**2.62 (1.52-4.49)**
**ARDS**				
Model 1	0.28 (0.04-2.07)	1.00	0.80 (0.35-1.82)	**3.44 (2.00-5.94)**
Model 2	0.23 (0.03-1.85)	1.00	0.70 (0.29-1.73)	**3.16 (1.69-5.88)**
**Death**				
Model 1	**3.71 (1.27-10.79)**	1.00	0.57 (0.15-2.10)	2.50 (1.08-5.79)
Model 2	**3.83 (1.22-11.71)**	1.00	0.53 (0.14-2.00)	1.74 (0.73-4.21)

Model 1: unadjusted.
Model 2: adjusted for age, sex, neutrophil counts, lymphocyte counts, platelet counts, hs-CRP, cancer (yes/no), diabetes (yes/no), hypertension (yes/no), CVD (yes/no), and center.
We did a sensitivity analysis to investigate the associations of BMI with IMV therapy, ICU admission, development of ARDS, and death with additional adjustment for center, diabetes, hypertension and CVD. The HRs for all subgroups were generally comparable with the main analysis.
ARDS, acute respiratory distress syndrome. BMI, body mass index. CVD, cardiovascular disease. ICU, intensive care unit. IMV, invasive mechanical ventilation.
